# Dispersal limitation dominates the community assembly of abundant and rare fungi in dryland montane forests

**DOI:** 10.3389/fmicb.2022.929772

**Published:** 2022-09-27

**Authors:** Jianming Wang, Yin Wang, Mengjun Qu, Jingwen Li

**Affiliations:** ^1^School of Ecology and Nature Conservation, Beijing Forestry University, Beijing, China; ^2^Key Laboratory of Ecosystem Network Observation and Modeling, Institute of Geographic Sciences and Natural Resources Research, Chinese Academy of Sciences, Beijing, China

**Keywords:** dryland montane forest, biogeographic patterns, habitat niche, stochastic processes, abundant and rare fungi

## Abstract

The assembly mechanisms and drivers of abundant and rare fungi in dryland montane forest soils remain underexplored. Therefore, in this study, we compared the distribution patterns of abundant and rare fungi and explored the factors determining their assembly processes in a dryland montane forest in China. Stronger distance-decay relationships (DDRs) were found in abundant sub-communities than in rare sub-communities. In addition, abundant fungi exhibited greater presence and wider habitat niche breadth than rare fungi. Both the null model and variation partitioning analysis indicated that dispersal limitation and environmental selection work together to govern both abundant and rare fungal assembly, while dispersal limitation plays a dominant role. Meanwhile, the relative influence of dispersal limitation and environmental selection varied between abundant and rare sub-communities, where dispersal limitation showed greater dominance in abundant fungal assembly. Mantel tests demonstrated that soil pH and phosphorus played critical roles in mediating abundant and rare fungi assembly processes, respectively. Our findings highlight that the distinct biogeographic patterns of abundant and rare fungi are driven by different assembly mechanisms, and the assembly processes of abundant and rare fungi are determined by diverse ecological drivers in dryland montane forest soils.

## Introduction

Soil microbes play crucial roles in mediating ecosystem structure and processes, such as nutrient and material cycles (Mooshammer et al., 2014; Delgado-Baquerizo et al., 2016; de Sosa et al., 2018). Elucidating the fundamental assembly process driving microbial richness and composition is vital to predicting the response of ecosystems to global changes (Zhou and Ning, 2017). Indeed, most processes related to community assembly can be classified into two classes: stochastic and deterministic processes (Stegen et al., 2012; Zhou and Ning, 2017; Liu L. et al., 2021). Niche theory postulates that niche processes, including abiotic and biotic selection, determine community assembly (Fargione et al., 2003). In contrast, the neutral theory assumes that all individuals in communities are ecologically equivalent and that communities are regulated by neutral processes, such as dispersal limitation and ecological drift (Chase and Myers, 2011). Multiple ecological processes are generally believed to work together and drive community assembly (Stegen et al., 2013), whereas their relative roles depend on time and space (Stegen et al., 2012; Dini-Andreote et al., 2015).

Soil microbial communities are primarily dominated by a few abundant species, while a mass of other species (“rare biosphere”) have an extremely low abundance (Jia et al., 2018; Egidi et al., 2019). Rare and abundant microbial assembly processes are subjected to divergent controlling mechanisms (Liu et al., 2015; Gao et al., 2020). Owing to the difference in competition capacity and stress tolerance, rare and abundant microbes exhibit quite different biogeographic patterns (Jiao and Lu, 2020b). Hence, comparing the ecological distribution and assembly mechanisms of rare and abundant microbes may be a good way to better infer microbe-driven ecosystem functioning. To date, biogeographical studies on abundant and rare bacteria have been extensively conducted in diverse environments (Jiao et al., 2017; Gao et al., 2020; Hou et al., 2020). Compared with bacteria, fungi have a larger body size (Powell et al., 2015) and can decompose complex molecules from plant litter inaccessible to most bacteria (Boer et al., 2005; Romaní et al., 2006). A previous study has found that soil fungal communities are structured by dispersal limitation, whereas deterministic factors shape bacterial composition in drylands (Wang et al., 2017). Our previous study has demonstrated that abundant and rare bacteria exhibit distinct biogeographic patterns and assembly mechanisms in the dryland montane forest. Therefore, a comparison between abundant and rare fungi is vital to exploring soil microbial assembly processes. However, the difference in distribution patterns and assembly processes between rare and abundant fungi in dryland montane forests has been barely elucidated.

An open question in ecology is whether and how environmental factors regulate the balance among different assembly processes (Tripathi et al., 2018). In fact, the environmental moderators of soil microbial community assembly processes have been widely examined in numerous ecosystems (Delgado-Baquerizo et al., 2020; Liu L. et al., 2021; Ni et al., 2021). Studies have reported that community assembly processes of soil microbes are influenced by different environmental variables, such as aridity, temperature, salinity, soil pH, and nutrients (Zhang et al., 2019; Jiao and Lu, 2020a; Wan et al., 2021), and their relative influence depends on soil microbial taxa, ecosystem types, and inquiry scales. As a particular component of dryland ecosystems, dryland montane forests are mainly distributed in high-elevation regions. Compared with grasslands and deserts, dryland montane forests are characterized by higher nutrient and water availability and lower temperatures (Wang et al., 2021a). Moreover, dryland forests have been reported to be particularly sensitive to climate change and associated increases in water stress (Liu et al., 2013; Poulter et al., 2013). Our previous studies have observed that soil pH and temperature rather than aridity drive the assembly processes of abundant and rare bacteria in dryland montane forests (Wang et al., 2021b). More importantly, studies on agricultural ecosystems and wetlands have demonstrated that divergent environmental factors mediate the assembly processes of abundant and rare fungi (Jiao and Lu, 2020a; Wan et al., 2021). Therefore, exploring the foremost drivers of soil fungal assembly processes in dryland montane forests may provide new evidence for fundamental mechanisms generating and maintaining biodiversity in drylands. However, the relative influence of different environmental factors on abundant and rare fungal assembly processes remains unclear.

Here, we aim to compare the distribution patterns and assembly mechanisms of abundant and rare fungi in dryland montane forest soils and test how different environmental factors jointly drive the assembly processes of abundant and rare fungi. Hence, we collected 24 samples from major distribution regions of dryland montane forests in China and assessed soil fungal communities based on high-throughput sequencing data of ITS. We hypothesized that (1) abundant and rare fungal sub-communities have distinct distribution patterns and assembly mechanisms and (2) divergent environmental factors regulate the assembly processes of abundant and rare fungi.

## Materials and methods

### Study region and field sampling

According to the distribution range of forest habitat, we selected 24 sites from a mountain forest ecosystem in northern Xinjiang, China, during the peak of the growing season (July–August) in 2016 (Supplementary Figure 1). The study region covers more than 450,000 km^2^. Its general topography is characterized by two longitudinal mountain systems (i.e., the Tianshan Mountains and Altay Mountains) separated by a basin (the Junggar Basin). The climate is mainly arid or semi-arid, with high variability of precipitation and temperature. At each site, a 20 m × 20 m plot was established from the representative vegetation. After that, 15 soil cores were combined per plot, taken at depths of 0–10 cm, and then mixed into a composite sample. Then all composite samples were sieved through a 2 mm mesh and divided into two portions: one portion was stored in thermally insulated boxes (at 4°C) for determining soil physicochemical properties, and the other was stored at −20°C until DNA extraction.

### Soil and climate data

Soil physicochemical properties, including soil pH, total phosphorus (TSP, g/kg), total nitrogen (TSN, g/kg), total organic carbon (TOC, g/kg), soil available nitrogen (AN, mg/kg), moisture content (SM, %), and soil N: P and C: N ratios. SM was measured gravimetrically by drying at 105°C to a constant weight. Soil pH was measured at a soil-to-water ratio of 1:2.5. TOC was measured by the K_2_Cr_2_O_7_ oxidation method. TSN was measured by the Kjeldahl procedure. AN was measured by the alkali diffusion method. TSP was measured by the molybdenum blue method.

Climatic variables, including mean annual temperature (MAT) and mean annual precipitation (MAP), were extracted from the WorldClim global climate database using the geographic coordinates of each site (resolution: 1 km × 1 km).^1^ We then obtained annual potential evapotranspiration (PET) from CGIAR-CSI (with a resolution of 1 km × 1 km).^2^ The aridity index (AI) was estimated as the ratio of MAP to PET (AI = MAP/PET; UNEP, 1992).

### Molecular and bioinformatics analysis

Total fungal DNA was extracted from 0.5 g of well-mixed fresh soil samples using E.Z.N.A. soil DNA kits (OMEGA, USA) following the manufacturer’s instructions. The fungal internal transcribed spacer (ITS) region was amplified using universal primers ITS1F (5′-CTTGGTCATTTAGAGGAAGTAA-3′) and ITS2 (5′- TGCGTTCTTCATCGATGC-3′) (Gardes and Bruns, 1993). High-throughput sequencing was performed on an Illumina Miseq PE300 sequencing platform at Beijing Allwegene Tech, Ltd. (Beijing, China).

Fungal sequences > 200 bp with an average quality score > 20 and without ambiguous base calls were quality processed within the QIIME package (Version 2.0). After that, the remaining high-quality sequences were clustered into operational taxonomic units (OTUs) using a 97% similarity threshold within UPARSE. The taxonomy of each ITS gene sequence was analyzed by comparison with sequences within the UNITE database (Version 8.2). OTUs were picked at 97% sequence similarity. Meanwhile, OTUs with reads of less than 20 were discarded to avoid the random influence on the identification of rare taxa (Jiao and Lu, 2020a). To eliminate the influence of sequencing depths on the analyses, sequences were rarefied at 21,042 sequences from each sample. In this study, OTUs with relative abundances above 0.1% of the total sequences were regarded as abundant, while those with relative abundances below 0.01% were defined as rare. Soil fungal raw sequences used in this paper are available in the NCBI Sequence Read Archive under BioProject PRJNA825059.

### Statistical analyses

Firstly, 11 environmental variables (MAT and AI for climate; SM, TSN, TOC, SAN, TSP, CN, NP, and pH for soil attributes; Altitude) were used in this study. To reduce strong collinearity between variables, we removed TOC and NP according to Pearson’s > | 0.7| (Supplementary Figure 2). Geographic distance matrices were calculated based on GPS coordinates, and then standardized environmental Euclidean distance matrices were calculated within the “vegan” package (Oksanen et al., 2015). The Bray-Curtis community dissimilarity distance was estimated to reflect the variance in species composition (β-diversity) among soil fungal communities. The slope of ordinary least-square regression between compositional similarity (1-β-diversity) and geographic distance was further used to quantify the distance–decay relationships (DDRs).

Levins’ niche breadth (B) index was employed to elucidate the patterns of stochastic and deterministic processes and their effects on soil fungal communities (Levins, 1968). The *B*-value of each fungal OTU was calculated following the previous study (Jiao et al., 2020). A higher *B*-value indicates a wider habitat niche breadth. Community *B*-values (*Bcom*) were quantified by abundance-weighted mean *B*-values from all fungal OTUs occurring within each community (Wu et al., 2018). A fungal community with a higher *B*-value is expected to be more metabolically flexible (Pandit et al., 2009). Notably, the “niche. width” function of the “spaa” R package was applied to calculate Levins’ niche breadth (B) index.

Abundance-based null model and neutral model analyses were used to infer the influence of ecological processes on soil fungal assembly (Kraft et al., 2011; Myers et al., 2013; Ning et al., 2019). In brief, 999 null local communities were generated by randomly resampling individuals into a local community with probabilities proportional to the regional abundance of the species while maintaining the same species richness and abundance (Ning et al., 2019; Liu W. et al., 2021). Afterward, the standardized effect size (β-deviation) of β-diversity was calculated using the following formula: β-deviation = [β-diversity_*obs*_ − Mean (β-diversity_*null*_)]/standard deviation (β-diversity_*null*_), where β-diversity_*null*_ and β-diversity_*obs*_ can denote the mean Bray–Curtis dissimilarity of null communities and observed β-diversity, respectively. Stochastic processes dominate community assembly if the β-deviation is statistically indistinguishable from zero; otherwise, the β-deviation remarkably greater than zero indicates a dominant influence on dispersal limitation of heterogeneous selection. Conversely, the domination of homogenizing dispersal or homogeneous selection would be supported if the β-deviation is significantly less than zero (Zhang et al., 2020). The null-model analysis was performed using “tNST” within the NST package (Ning et al., 2019). Additionally, the null-model approach conducted based on phylogenetic β-diversity can better evaluate the relative roles of different assembly processes (Stegen et al., 2013). However, fungal ITS is a variable region and cannot be aligned, so this study did not implement such analyses (Zinger et al., 2019). Meanwhile, the contribution of stochastic processes was further calculated using a neutral model by predicting the association between abundance and frequency of taxonomic occurrence (Sloan et al., 2006). *R*^2^ indicates the fit to the neutral model. The neutral model analysis was performed using the “snm” function within the iCAMP package (Ning et al., 2020).

To further identify the relative roles of environmental and dispersal limitation, we partitioned the relative influence of spatial and environmental factors on β-deviations through variation-partitioning analysis (VPA), which was performed using the “vegan” package in R. Multiple regressions on distance matrices (MRMs) were used to select environmental and spatial factors through forwarding selection until *P* < 0.05. The MRM test was performed using the “ecodist” package in R. The individual influence of spatial factors represents the effect of dispersal limitation, whereas the individual effect of environmental distance indicates the importance of environmental selection (Myers et al., 2013; Zhang et al., 2020). After that, the effect ratio of environmental selection to dispersal limitation (ESDS) was used to elucidate further the relative importance of environmental selection and dispersal limitation. Finally, the Mantel test was conducted to elucidate the influence of different environmental factors on the relative importance of different assembly processes.

## Results

### General distribution patterns of abundant and rare taxa

**FIGURE 1:**
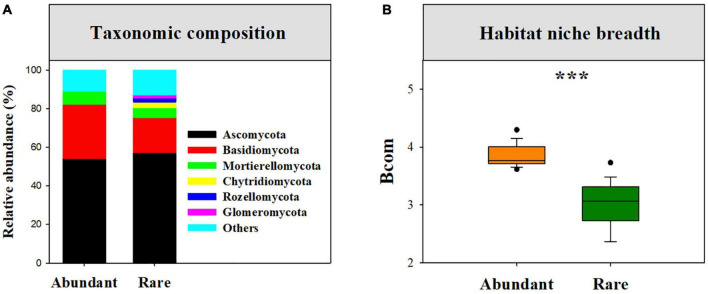
The taxonomic composition of rare and abundant fungal sub-communities **(A)**, and the difference in mean habitat niche breadths (*Bcom*) between abundant and rare fungal subcommunities **(B)**, and ***P < 0.0001; Wilcoxon rank-sum test.

After quality filtering and removing chimeric sequences, 505,008 high-quality sequences were clustered into 1,688 OTUs. Across those fungal OTUs, a total of 969 OTUs (57.41%) with 23,647 sequences (4.68%) were identified as rare fungi, while only 172 OTUs (10.19%) with 398,367 sequences (78.88%) were identified as abundant fungi (Supplementary Table 1). Abundant sub-communities were mainly dominated by *Ascomycota* (54.08%), *Basidiomycota* (27.17%), and *Mortierellomycota* (6.82%), whereas rare sub-communities were primarily dominated by *Ascomycota* (57.30%), *Basidiomycota* (18.17%), *Mortierellomycota* (5.03%), *Chytridiomycota* (3.05%), *Rozellomycota* (2.04%), and *Glomeromycota* (1.59%) (Figure 1A).

**FIGURE 2:**
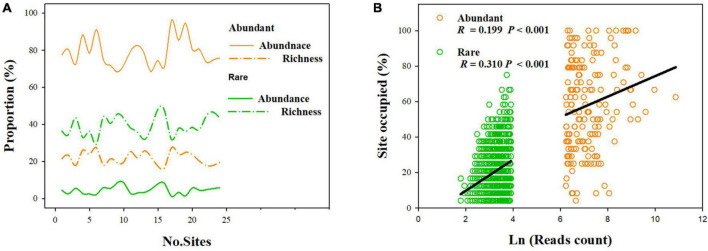
Distributions of rare and abundant fungi in desert soils. **(A)** The proportion of the OTUs’ richness (“richness”) and relative abundance of abundant and rare fungi compared to the whole fungal community in each sample. **(B)** Abundance–occupancy relationships for the abundant and rare sub-communities.

**FIGURE 3:**
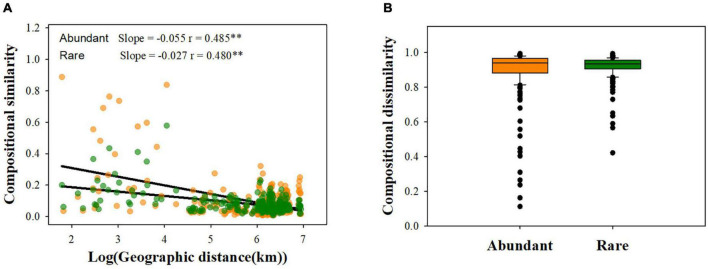
Patterns of rare and abundant fungal β-diversity. **(A)** Distance- decay curves of community similarity for rare and great fungi; **(B)** fungal β-diversity between rare and abundant subcommunities. ^**^P < 0.01.

Our results showed that abundant fungi had a greater presence than rare fungi across soil samples. Specifically, 62.21% of abundant OTUs occurred in > 50% of samples, whereas only 3.20% of the rare OTUS (31 OTUs) were present in > 50% of samples (Figure 2A). Abundance–occupancy relationships indicated that abundant fungi showed weaker positive associations than rare fungi (Figure 2B). Meanwhile, we observed remarkably higher mean *Bcom* values in abundant sub-communities than in rare sub-communities (Figure 1B). Remarkable DDRs between geographic distance and community similarity were found in both abundant and rare sub-communities (*P* < 0.001, Figure 3A), and the slope of DDRs was much stronger in abundant sub-communities than in rare sub-communities. Furthermore, abundant and rare sub-communities did not differ significantly in community β-diversity (Figure 3B). Both the species composition of abundant fungi was mainly shaped by spatial factors, followed by SM and TSN (*R*^2^ = 0.311 and 0.308, Supplementary Table 2).

### Assembly processes of rare and abundant fungal sub-communities

**FIGURE 4:**
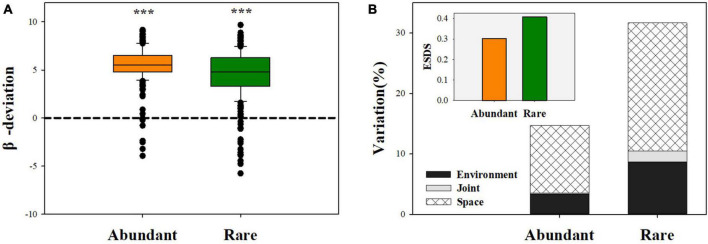
The β-deviations for abundant and rare fungal subcommunities. **(A)** Difference between abundant and rare fungal β-deviation and 0 values; **(B)** variation in β-deviations were explained by spatial and environmental variables after forward-model selection. ***P < 0.0001; Wilcoxon rank-sum test.

The neutral community model explained a larger fraction of the variation in the abundant sub-community (*R*^2^ = 0.87) than in the rare sub-community (*R*^2^ = 0.59; Table 1). The null model analysis showed that β-deviations for both abundant and rare fungal subcommunities were significantly greater than zero (Figure 4A), implying the dominance of dispersal limitation or heterogeneous selection. VPA showed that environment and space explained the total amount of variation in rare fungal β-deviations than in abundant β-deviations (Figure 4B). Environmental and space individually explained 3.40 and 11.2% of the variation in abundant fungal β-deviation, with an ESDR of 0.30. Meanwhile, environmental and space individually explained 8.67 and 21.23% of the variation in rare fungal β-deviation, with an ESDR of 0.408. These results showed that both abundant and rare fungal assembly were mainly regulated by dispersal limitation, while dispersal limitation played a relatively more important role in the abundant fungal assembly.

**TABLE 1:** Fit of the neutral model in abundant and rare fungal sub-communities in dryland montane forest soil.

Abundant		Rare	
	
*M*	*R* ^2^	*M*	*R* ^2^
0.29	0.87	0.86	0.59

**FIGURE 5:**
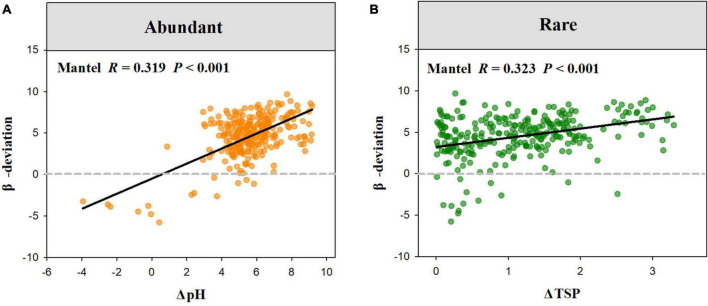
The relationships between abundant **(A)** and rare **(B)** fungal β-deviation and difference in pH and TSP.

The Mantel test showed that β-deviation of abundant sub-communities was significantly related to the differences in MAT, soil nutrient, and pH, while that of rare sub-communities was significantly associated with differences in altitude, MAT, AI, soil nutrient, and pH (Table 2). Among these environmental factors, the β-deviation of abundant sub-communities was more influenced by the difference in pH, while that of rare sub-communities was more influenced by the difference in TSP. Increasing differences in soil pH and phosphorus resulted in increased stochasticity for abundant and rare sub-communities, respectively (Figure 5).

**TABLE 2:** Mantel test results showing internal links of β-deviation and environmental and spatial distances.

Variables	Abundant		Rare	
		
	Mantel *R*	*P*	Mantel *R*	*P*
Space	0.336	<0.001	0.480	<0.001
SM	0.052	>0.05	−0.031	>0.05
TSN	0.187	<0.05	0.029	>0.05
TSP	0.191	<0.05	**0.323**	**<0.001**
CN	0.232	<0.05	0.178	<0.01
AN	−0.141	>0.05	0.046	>0.05
pH	**0.319**	**<0.001**	0.201	<0.01
MAT	0.258	<0.01	0.192	<0.05
AI	−0.018	>0.05	0.209	<0.01
Altitude	0.107	>0.05	0.177	<0.01

Values in bold indicate relative stronger correlation (Mantel R) in abundant and rare fungal subcommunities.

## Discussion

### Differential distribution and environment preference of abundant and rare fungi

Understanding species distribution patterns and ecological preferences is critical for predicting how species respond to ongoing environmental changes (Maharjan et al., 2021). Consistent with the findings reported for the whole fungal community (Li et al., 2021), robust DDRs were found for abundant and rare fungi. But the steeper distance-decay slope of abundant fungi indicated that the turnover rate of abundant fungi was considerably faster than that of rare fungi (Figure 3A). The divergence in distribution patterns of abundant and rare fungi may be attributed to differences in dispersal potential and tolerance capability. We also found narrower habitat niche breadth and less ubiquity for rare fungi than abundant fungi (Figure 1B and Figure 2), indicating that rare taxa have lower tolerance and adaptability to harsh environments than abundant taxa (Delgado-Baquerizo et al., 2018). This phenomenon may reflect that rare taxa are ill-suited to most desert habitats (Brown, 1984) and therefore are limited by habitat specificity (Barberán et al., 2014; Jousset et al., 2017). Taken together, these findings reveal differential distribution patterns of rare and abundant fungi in dryland montane forests.

### Dominant role of dispersal limitation in abundant and rare fungal assembly

Disentangling the relative contributions of deterministic and stochastic processes to microbial soil assembly can help better infer microbially driven ecosystem processes and functions (Nemergut et al., 2013). In this study, the neutral model analysis indicated that abundant sub-communities were more affected by neutral processes (Table 1). Both the null model and VPA analysis further demonstrated that dispersal limitation and environmental selection work together to govern both soil abundant and rare fungal assembly, whereas dispersal limitation showed a dominating effect on both abundant and rare fungal assembly (Figure 4). Meanwhile, our results also revealed that dispersal limitation has a greater relative contribution in abundant fungal assembly than in the rare, which supports prior reports that abundant sub-communities are more limited by dispersion than rare sub-communities (Wu et al., 2017; Jiao and Lu, 2020a). Most abundant species are more prone to dispersal limitation because more individuals can potentially be involved in a dispersal event (Liu et al., 2015). Moreover, it is noteworthy that a large proportion of the variation in fungal β-deviations remained unexplained by selected environmental and spatial factors (Supplementary Table 2). This result was consistent with the findings of previous studies, which may reflect the influence of other unidentified biotic factors (i.e., plant litter or plant traits; Yang et al., 2019; Guo et al., 2020; Wang et al., 2022). Together, these results implied that dispersal limitation played a greater role than environmental selection in shaping the community assembly of abundant and rare fungi.

More importantly, our results indicated that environmental selection had a stronger influence on a rare fungal assembly than the abundant. It is widely believed that abundant species occupy diverse niches and have higher resource competitiveness and greater tolerance and adaptability to environmental changes than rare species (Kraft and Ackerly, 2010; Delgado-Baquerizo et al., 2018). Hence, a rare fungal assembly is more easily influenced by environmental selection than an abundant one. Additionally, our results are also inconsistent with previous reports that environmental selection dominates in rare fungal sub-communities in agricultural and apple orchard soil (Jiao and Lu, 2020a; Zheng et al., 2021), probably due to the difference in environmental regime and geography among studies (Chase, 2010; Zhou et al., 2014). Taken together, our findings reveal dominant roles for stochastic processes in abundant and rare fungal assembly.

### Soil pH and phosphorus drove the variation in the assembly process of abundant and rare fungi in dryland montane forests

Uncovering drivers mediating the balance between deterministic and stochastic processes in soil microbial communities is vital to gaining an advanced mechanistic understanding of microbial ecology (Feng et al., 2018; Tripathi et al., 2018). Previous studies have reported that community assembly processes of soil microbes are regulated by a wide range of environmental factors, such as soil pH, salinity, nutrients, and temperature (Shen et al., 2019; Zhang et al., 2019; Jiao and Lu, 2020a,b; Ni et al., 2021). In this study, we found both the difference in soil pH, nutrients, MAT, and AI were significantly related to the variation in the balance between different assembly processes of abundant and rare fungi. However, the assembly process of abundant and rare fungi was more affected by soil pH and phosphorus (STP). Increasing differences in soil pH and STP resulted in increased stochasticity for abundant and rare sub-communities, respectively (Figure 5).

**FIGURE 6:**
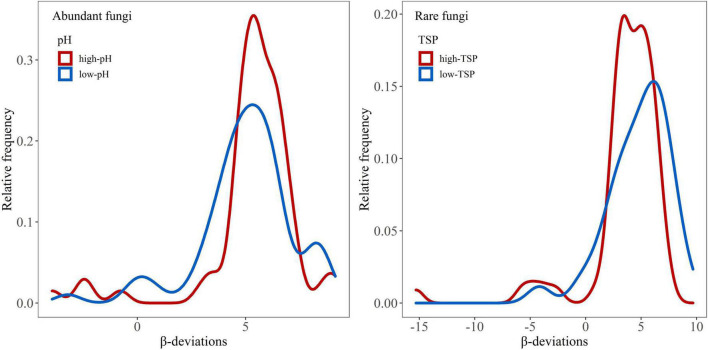
β-deviations distributions of abundant and rare fungi in different soil pH and STP gradients, respectively. These two categories (i.e., high-pH vs. low-pH) were divided by standardizing the number of samples in each category, which is more reasonable when comparing distinct categories (Jiao et al., 2021).

Soil pH and nutrients are the key determinants of ecosystem structure and processes at multiple scales (Förstner, 1994; Xu et al., 2018; Neina, 2019; Qin et al., 2020). We further found that the relative frequency of abundant fungal β-deviation in high-pH sites (pH 6.7–7.5) was higher than in low-pH sites (pH 4.3–6.3) (Figure 6), which indicated that the relative importance of dispersal limitation on abundant fungi was higher in neutral soil than weakly acid soils. Neutral soils were suitable for most soil microbes due to their weakened environmental stress and selection strength in them (Tripathi et al., 2018), which may induce the increased role of dispersal limitation in high-pH sites (neutral soil). Furthermore, we observed that the relative frequency of rare fungal β-deviation in high-TSP sites (TSP 0.71–1.04) was higher than in low-TSP sites (TSP 0.36–0.70) (Figure 6), which demonstrated that dispersal limitation on rare fungi was more important in high-TSP sites. The increased role of dispersal limitation in high-STP sites may be owing to higher nutrient availability that could enhance the ability of rare fungi to disperse, which is inconsistent with the resource supply–stochasticity relationships (Dini-Andreote et al., 2015). Together, these findings revealed that the relative influence of environmental selection and dispersal limitation on abundant and rare fungi in dryland montane forests was driven by variations in pH and STP, respectively.

## Conclusion

This study compared abundant and rare fungi distribution patterns and assembly mechanisms in dryland montane forests along wide environmental gradients. Abundant and rare fungal community similarities showed different relationships with geographic distance. Abundant fungi exhibited greater presence and wider habitat niche breadth than rare fungi. Dispersal limitations of stochastic processes dominated abundant and rare fungal sub-communities, whereas they exerted relatively greater effects on abundant fungal sub-communities. Soil pH and phosphorus played critical roles in mediating the assembly processes of abundant and rare fungi, respectively. Our study highlights the distinct distribution patterns and assembly mechanisms of abundant and rare fungal sub-communities and reveals that the assembly processes of abundant and rare fungi are determined by diverse ecological drivers in dryland montane forest soils.

## Data availability statement

The datasets presented in this study can be found in online repositories. The names of the repository/repositories and accession number(s) can be found below: https://www.ncbi.nlm.nih.gov/, PRJNA825059.

## Author contributions

JW and JL designed the study. JW and YW performed the field investigation and collected the data. JW and MQ developed the methods. JW, YW, MQ, and JL wrote the manuscript.

All authors contributed to the article and approved the submitted version.

## Footnotes

1
http://www.worldclim.org

2
https://cgiarcsi.community/category/data/
