# Supplementary material for: Dispersal limitation dominates the community assembly of abundant and rare fungi in dryland montane forests
Source: Front Microbiol. 2022 Sep 27;13:929772. doi: 10.3389/fmicb.2022.929772 (PMC9551450; doi:10.3389/fmicb.2022.929772)
Supplement: Supplementary file 1 [file Data_Sheet_1.doc]

**Dispersal limitation dominate the community assembly of abundant and rare fungi in** **dryland montane forests**

Jianming Wang^1,2^, Mengjun Qu^1^, Yin Wang^1^, Jingwen Li^1^*

1. School of Ecology Nature Conservation, Beijing Forestry University, Beijing, China.

2. Key Laboratory of Ecosystem Network Observation and Modeling, Institute of Geographic Sciences and Natural Resources Research, Chinese Academy of Sciences, Beijing 100101, China

* Corresponding author

Table S1 General description of all, abundant and rare OTUs data sets

|  | OTU numbers | Sequence numbers |
| --- | --- | --- |
| All OTUs | 1,688 | 505,008 |
| Abundant OTUs | 172 (10.19%) | 398,367 (78.88%) |
| Rare OTUs | 969 (57.41%) | 23,647 (4.68%) |

OTUs with relative abundances above 0.1% of the total sequences were regard as abundant, while those with relative abundances below 0.01% were defined as rare

Table S2 Multiple regressions on distance matrices of the community compositions for abundant and rare fungal sub-communities with spatial and environment variables.

|  | Variables | Slope | *P* | Model *R*^2^ | Model *P* |
| --- | --- | --- | --- | --- | --- |
| **Abundant** | Space | 0.052 | <0.01 | 0.311 | <0.001 |
|  | SM | 0.018 | <0.05 |  |  |
|  | TSN | 0.033 | <0.01 |  |  |
|  |  |  |  |  |  |
| **Rare** | Space | 0.026 | <0.01 | 0.308 | <0.001 |
|  | SM | 0.012 | <0.05 |  |  |
|  | TSN | 0.014 | <0.01 |  |  |


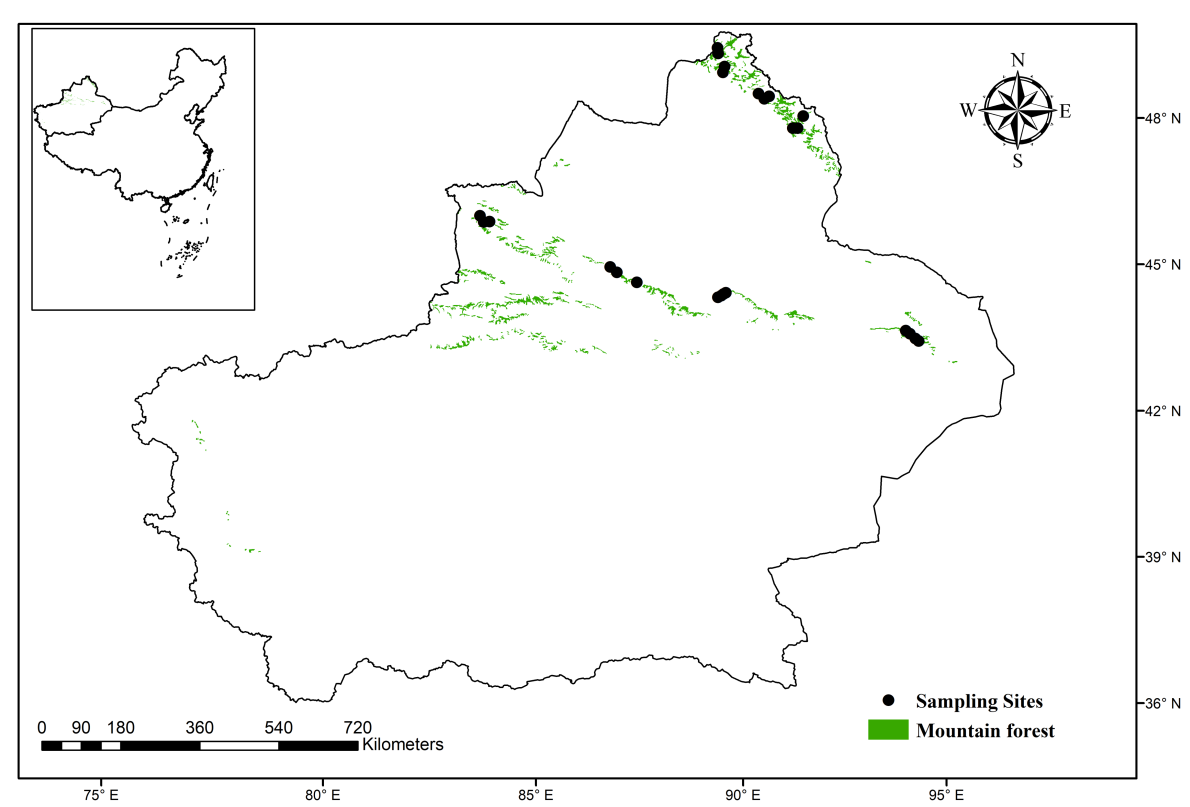


**Figure S1** Locations of the sampling sites for this study and the distribution map of dryland montane forest ecosystems in northwest China.


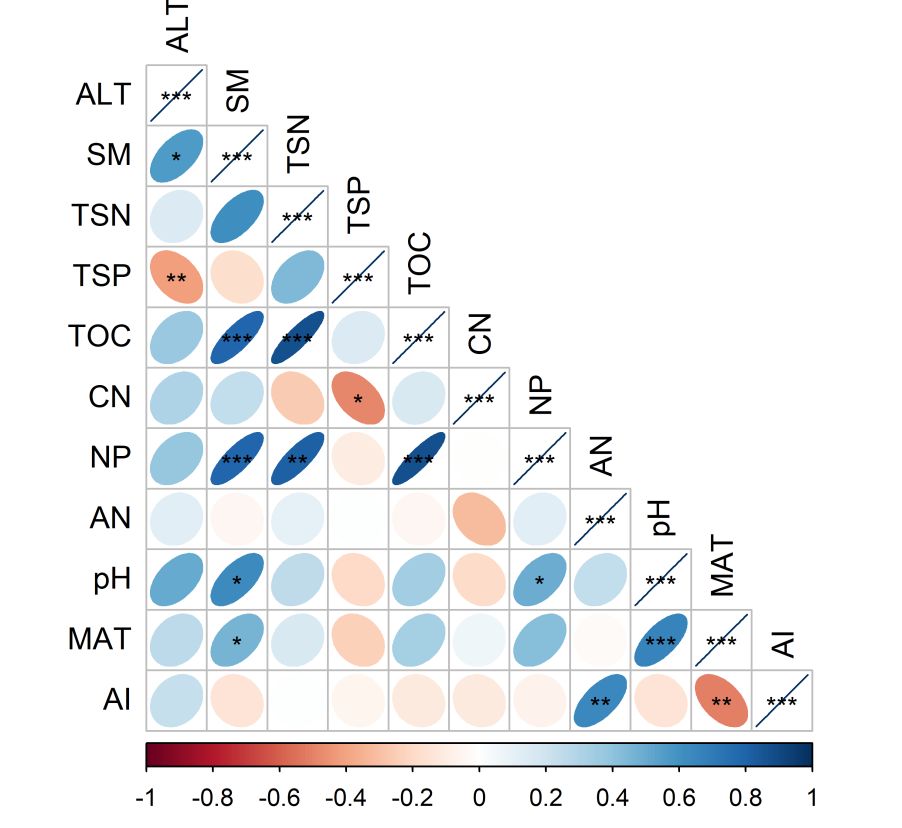


**Figure S2** Correlations among soil and climatic variables in dryland montane forests. pH, soil pH; TSP, soil total phosphorus; TSN, soil total nitrogen; TOC, soil total organic carbon; AN, soil available nitrogen; SM, moisture content; NP and CN, soil N: P and C: N ratios; MAT, mean annual temperature; AI, aridity index.
